# Are redo urethroplasties prone to failure? Objective success rates and patient satisfaction in recurrent urethral stricture repair

**DOI:** 10.1590/S1677-5538.IBJU.2026.0081

**Published:** 2026-06-30

**Authors:** Yishai H. Rappaport, Sarit Cohen, Boris Chertin, Ofer Z. Shenfeld

**Affiliations:** 1 Hebrew University school of medicine Center for reconstructive and functional urology Shaare Zedek medical center Jerusalem Israel Center for reconstructive and functional urology, Shaare Zedek medical center, the Hebrew University school of medicine, Jerusalem, Israel; 2 Ben Gurion University Goldman school of medicine Department of Urology Beer-Sheba Israel Department of Urology, Samson Assuta Ashdod medical center, the Goldman school of medicine, Ben Gurion University, Beer-Sheba, Israel; 3 Hebrew University school of medicine Shaare Zedek medical center Department of Urology Jerusalem Israel Department of Urology, Shaare Zedek medical center, the Hebrew University school of medicine, Jerusalem, Israel

**Keywords:** Urethral Stricture, Surgical Procedures, Operative, Recurrence, Patient Reported Outcome Measures

## Abstract

**Objective:**

To evaluate long term success rates and patient reported outcomes of redo urethroplasty (RU), a technically challenging urological procedure that may be associated with lower success rates due to increased scarring, compromised blood supply and poor surgical planes from prior surgery.

**Materials and methods:**

We retrospectively reviewed RU patients between 2010–2023, excluding failed hypospadias repair, and compared with a cohort of primary urethroplasty patients. Failure was defined as need for urethral intervention or imaging/cystoscopy-confirmed stricture recurrence prompted by symptoms. Primary endpoint was stricture-free survival. Secondary endpoints included patient-reported outcomes (USS-PROM), satisfaction, and subgroup analysis by stricture location. Statistical tests included T-tests, Wilcoxon signed-rank, and Fisher's exact test.

**Results:**

Sixty redo urethroplasty patients (median age 48.5) were compared to 316 primary cases (median age 45, p=0.84). Median follow-up was 35 (IQR 14-81) vs. 51 (IQR 20-114) months for redo and primary cases, respectively (p=0.07). Failure rates were not significantly different: 23% in redo vs. 15% in primary cases (p=0.12). However, time to failure was significantly shorter in the redo group (median 6 vs. 17 months, p=0.01). USS-PROM questions 1–6 favored the primary group, although bother scores (Q7) were similar. Overall satisfaction was high in both groups but slightly higher after primary repair (95% vs. 87%, p=0.04).

**Conclusion:**

Despite greater complexity, redo urethroplasty demonstrates similar long-term success rates to primary urethroplasty. Patient-reported bother was similar, supporting redo urethroplasty as a viable option for recurrent urethral stricture after previous repair. However, recurrences occurred significantly earlier in the redo group.

## INTRODUCTION

Although Urethroplasty is considered the gold standard treatment for urethral strictures, recurrence remains a significant issue. This has been observed in long-term follow up (15 years), in 14-58% of patients, depending on stricture location, pathogenesis and the reconstructive technique used (1). Management of recurrent strictures varies widely among urologists with standardized guidelines lacking (2). Direct vision internal urethrotomy (DVIU) is a reasonable option mostly for short recurrent strictures (≤ 1 cm) (3, 4) and has been shown to be superior to urethral dilation (5). However, approximately half of these patients experience further recurrence, highlighting the limitations of endoscopic management. Newer endoscopic options such as drug coated balloons have been recently reported, however post-urethroplasty strictures were generally excluded (6).

Redo urethroplasty is technically demanding due to scarring, loss of normal surgical planes, reduced vascularity and spongiofibrosis (7). Several studies have reported success rates for repeat urethroplasty, with a minority directly comparing primary to redo procedures (7-10). These reports generally show comparable stricture free rates, although definitions of success varied. We present our experience regarding success rate of primary vs. redo urethroplasty using a stringent definition of stricture free survival and unique to this study, patient reported outcomes, as recorded by the USS-PROM questionnaire (11-13). We hypothesized that redo cases would demonstrate inferior objective and subjective outcomes.

## MATERIALS AND METHODS

This retrospective study analyzed data from our prospectively maintained urethroplasty database, approved by the institutional review board (IRB 0155-19-SZMC). All patients undergoing redo urethroplasty between 2010 and 2023 were included. Redo urethroplasty was defined as urethroplasty performed for recurrent stricture after 1 or more failed previous urethroplasties, regardless of the number of previous dilations/endourethrotomies. A contemporaneous cohort of patients who underwent primary urethroplasty served as the comparison group.

Exclusion criteria included patients with failed hypospadias repairs, and those lacking post-operative follow up.

Pre-operative evaluation included history, physical examination, lab analysis (urine analysis and culture, creatinine levels) and a combination of uroflowmetry, post-void residual volume (PVR) in patients that did not present with urinary retention, retrograde urethrography (RUG) and voiding cystourethrography (VCUG). Cystourethroscopy was performed when indicated.

All procedures were performed by a single surgeon (O.Z.S).

Peri-operative complications (infection, extravasation, etc.) were recorded. Data were prospectively entered at the time of surgery in the center's urethroplasty database and updated at each follow up visit.

A urethral catheter and suprapubic tube (SPT) were routinely placed during surgery. Urethral catheter was removed and VCUG performed, 21-28 days post-operatively. In the absence of extravasation, the SPT was removed shortly thereafter. In case of extravasation repeated VCUG was performed 7-14 days later. The patients were seen at 1, 3, 6 and 12 months after the surgery, and annually thereafter. Follow up included a review of patient's symptoms, USS-PROM questionnaire, when possible, uroflowmetry and PVR. Cystourethroscopy was performed routinely 1 year after surgery and once more based on worsening symptoms, uroflowmetry, PVR, or PROM scores.

A stringent definition of surgical failure was used: either the need for additional urethral intervention due to stricture recurrence (reduced urinary stream or urinary retention), the demonstration of stricture on imaging (retrograde urethrography) or cystourethroscopy (inability to advance 16Fr flexible cystoscope).

The primary endpoint was absence of stricture recurrence. Secondary endpoints included patient satisfaction and USS-PROM questionnaire scores.

T-test and Wilcoxon signed-rank tests were conducted for continuous data and Fisher's exact test for categorical data. Statistical significance was defined as p < 0.05.

## RESULTS

Sixty patients underwent redo urethroplasty between 2010 to 2023 and were compared with 316 primary urethroplasty cases. Median age at surgery was 48.5 years (IQR 32-61) in the redo urethroplasty group and 45 years (IQR 32-61) in the primary group (p=0.84). Median follow up was 35 months (IQR 14-81) and 51 months (IQR 20-114), respectively (p=0.07). Stricture length, pre-op maximal urinary flow (Qmax), PVR, rates of urinary retention and urethral rest with suprapubic drainage preoperatively did not differ between groups. Baseline demographic, clinical parameters and stricture location are presented in Table-1.

**Table 1:** Patient characteristics, stricture features, and outcomes of redo versus primary urethroplasty.

Parameter		Redo urethroplasty (N=60)		Primary urethroplasty (N=316)		P value
		**Mean (SD)/Median (IQR)**	**Min, Max**	**Mean (SD)/Median (IQR**	**Min, Max**	
**Patient characteristics**						
	Age (years)	48.5 (32-61)	7-75	45 (30-62)	5-93	0.84
	Follow-up (months)	35 (14-81)	1-202	51 (20-114)	1-264	0.07
**Pre-operative parameters**						
	Qmax (mL/s)	3.7 ± 4.0	0-13	3.8 ± 4.1	0-16	0.86
	PVR (mL)	250 ± 309	0-700	160 ± 191	0-2000	0.003
	Urethral rest with suprapubic catheter, n (%)	18 (30)		110 (35)		0.55
**Stricture characteristics**						
	Stricture length (cm)	4.5 ± 3.9	1-18	5.2 ± 4.3	1-23	0.24
**Stricture location, n (%)**						
	Bulbar	23 (38)		211 (67)		<0.001
	Membranous	12 (20)		35 (11)		0.09
	Pan-anterior	13 (22)		36 (11)		0.04
	Penile/penobulbar	12 (20)		34 (11)		0.05
**Post-operative outcomes**						
	Good VCUG at 3–4 weeks, n (%)	55 (92)		304 (96)		0.12
	Qmax at 1 month (mL/s),	15.6 ± 10.4	0-51	17.7 ± 8.4	0-47	0.09
	PVR at 1 month (mL)	11.5 ± 28.2	0-150	16.9 ± 47.5	0-500	0.39
	Stricture recurrence at 12 months, n (%)	9 (15)		16 (5)		0.009
**Last follow-up**						
	Qmax (mL/s)	16.8 ± 12.1	0-64	18.6 ± 10.5	0-50	0.23
	PVR (mL)	96.9 ± 135.2	0-700	85.7 ± 126.6	0-700	0.53
	Stricture recurrence, n (%)	14 (23)		47 (15)		0.12
	Time to recurrence (months)	6 (2-11)	1-79	17 (6-52)	2-147	0.01
**Patient-reported outcomes**						
	USS-PROM sum questions 1-6	6.5 ± 5.4	0-18	5.7 ± 4.6	0-21	0.23
	Satisfaction with surgery, n (%)	52 (87)		300 (95)		0.04
	IIEF-5 score	18.6 ± 7.5	5-25	19 ± 8	0-25	0.72

IQR = Interquartile range; PVR = post-void residual volume; Qmax = maximum urinary flow rate; VCUG = voiding cystourethrography; USS-PROM = Urethral Stricture Surgery Patient-Reported Outcome Measure; IIEF-5 = International Index of Erectile Function-5.

Stricture location differed significantly, with 67% of primary patients and 38% of redo patients having a bulbar urethral stricture (p=0.0001). Intra and post-operative complication rates were similar (p=0.82) and all complications were considered minor (Clavien-Dindo 1-3).

Stricture recurrence occurred in 14 redo patients (23%) and 47 (15%) primary patients (p=0.12). However, time to stricture recurrence was significantly shorter after redo urethroplasty (median 6 months, IQR 2-11) vs. 17 months (IQR 6-52) in primary cases (p=0.01; Figure-1). This was also reflected in the higher recurrence rate at the 12-month time point.

**Figure 1:**
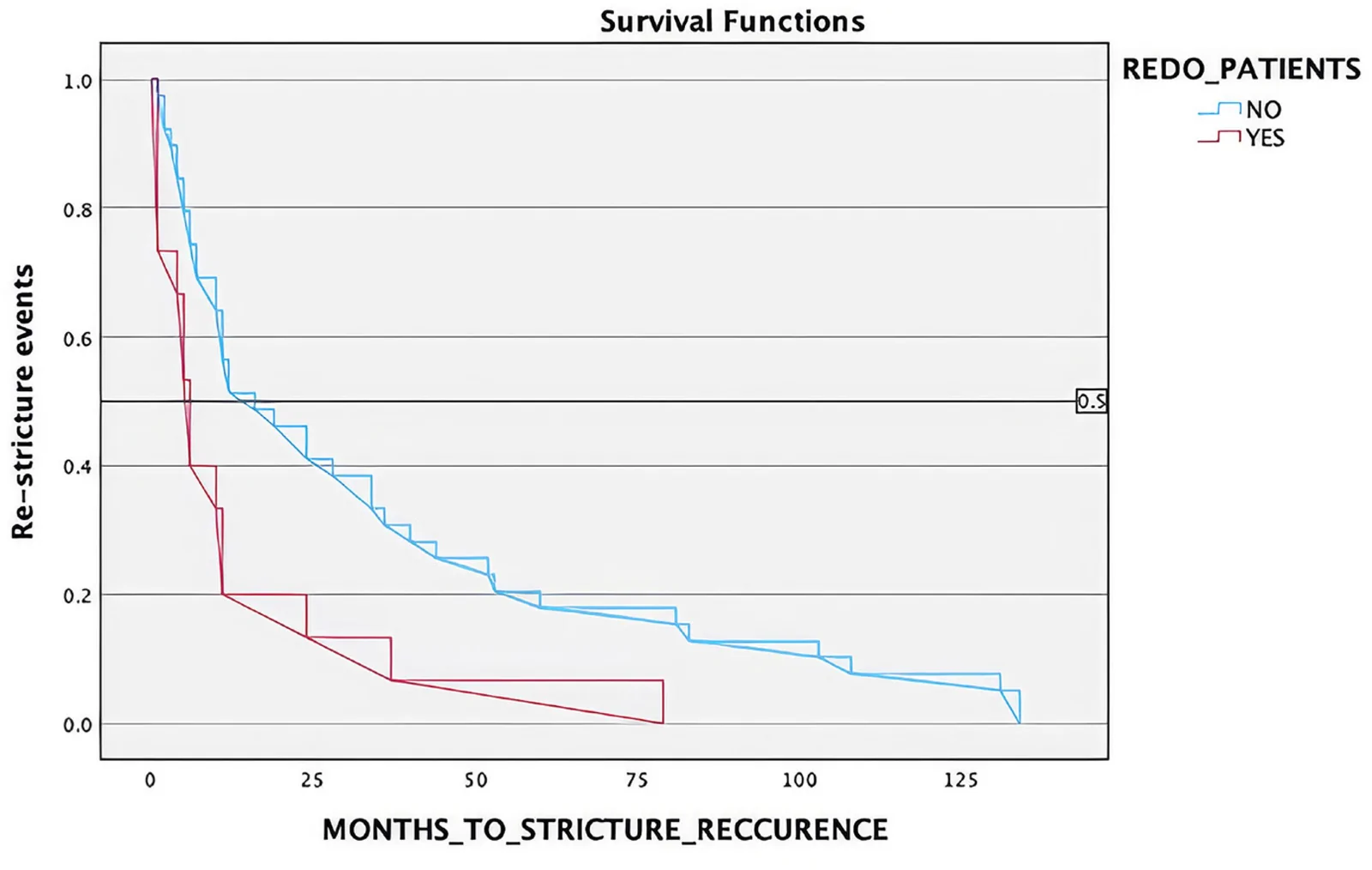
Kaplan-Meier survival curves for re-stricture rates after redo urethroplasty (red, n=60) and primary urethroplasty (blue, n=316) for anterior urethral strictures.

Re-stricture events were defined as either worsening urinary symptoms requiring intervention or stricture non-accommodating a flexible 16Fr cystoscope. Months to stricture recurrence was defined as the time from surgery to recurrent urethral stricture or last follow-up. The log-rank test was used to compare groups (p=0.13). Time to stricture recurrence was shorter after redo urethroplasty (p=0.01).

Post-operative Qmax (18.6 ± 10.5 vs. 16.8 ± 12.1, p=0.23) and PVR (85.7 ± 126.6 vs. 96.9 ± 135.2, p=0.53) were comparable.

The sum score of questions 1 through 6 in the USS-PROM questionnaire and the bother scores (Q7) did not differ between the groups. Overall satisfaction was high for both groups though higher in the primary group (95% vs. 87%, p=0.04). Erectile function (IIEF-5) scores did not differ between the primary (19 ± 8) and redo groups (18.6 ± 7.5, p=0.72).

Surgical techniques used are shown in Table-2.

**Table 2:** Surgical techniques used for redo urethroplasty and primary urethroplasty.

		Redo urethroplasty (n=60)	Primary urethroplasty (n=316)	P-value
**Anastomotic repair**		23 (38%)	143 (45%)	0.4
**Substitution**	Buccal mucosa graft	18 (30%)	132 (42%)	0.11
	Sublingual graft	2 (3%)	-	0.03
	Skin flap	2 (3%)	14 (4%)	>0.99
**Staged repair**		5 (8%)	11 (3%)	0.15
**Multiple techniques**		6 (10%)	40 (13%)	0.67
**Perineal urethrostomy**		12 (20%)	19 (6%)	0.001

In several cases more than one surgical technique was used concomitantly, causing sum of procedures to be more than 100%.

Subgroup analysis of by stricture location was underpowered; however, results suggest no significant differences with p=0.08, p=0.1, p=0.35 and p=1 for bulbar, membranous, pan-anterior and penile/peno-bulbar strictures, respectively.

## DISCUSSION

Redo urethroplasty is challenging due to several factors including scarring, lack of clear surgical planes, compromised vascularity and spongiofibrosis. Thus, surgeon knowledge and competency in numerous reconstructive techniques is required.

Redo urethroplasty success rates have varied in the literature while often been indirectly compared to primary urethroplasty results (3, 7, 9, 10). Furthermore, defining surgical failure and success has also been inconsistent across reports. Direct comparisons have been described (8, 10, 14) demonstrating redo cases to be feasible with results slightly favoring primary urethroplasty. Patient satisfaction can therefore be expected to correlate with stricture free survival.

Stricture location influences recurrence risk. Our findings align with previous reports demonstrate an increased risk of stricture recurrence for penile and pan-anterior strictures (15, 16). Possible contributing factors may include presence of Lichen Sclerosus, predominantly located in the penile urethra that is associated with higher failure rates, and the reliance on more complex substitution reconstructive techniques (17-19). Indeed, lichen sclerosus was present in (12%) of redo cases whereas only in 5% of primary patients. We excluded patients who had surgery for recurrent stricture after previous hypospadias repair due to the sparsity of data in our database regarding the number and type of previous hypospadias operations. Additionally, one may argue that hypospadias is a different disease process to urethral stricture disease, with a deficient corpus spongiosum, adding significant heterogeneity that may have confounded analysis. In our study, stricture location varied significantly between the groups, two thirds of primary urethroplasties were a bulbar urethral stricture whereas in only 38% of the redo patients (p=0.0001) was the stricture located in the bulbar urethra. Indeed, suggesting that non-bulbar strictures are at an increased risk for recurrence. We acknowledge this baseline imbalance as a potential source of confounding. However, sub-group analysis based on stricture location did not demonstrate significant differences in recurrence between redo and primary groups. The location imbalance would, if anything, bias against finding favorable outcomes in the redo cohort given the higher proportion of nun-bulbar strictures.

Surgical technique was chosen according to stricture location, length, complexity and the availability and quality of tissues. In primary cases, anastomotic repair and substitution repair using buccal mucosa graft tended to be more common. Perineal urethrostomy, was used more frequently in redo cases as appropriate for complex recurrent strictures. This reflects real-world case selection rather than bias; prior reports confirm comparable success and satisfaction with urethrostomy in select patients (20).

Patient satisfaction rates with redo urethroplasty were studied by Pfalzgraf et al. who demonstrated relatively high satisfaction and quality of life rates with acceptable rates of glans sensory alterations and subjective penile shortening (21). Results from our study are comparable to the literature, with high satisfaction rates to both primary (95% satisfied or very satisfied) and redo urethroplasties (87%), the difference reaching significance (p=0.04).

In support of overall patient satisfaction, erectile function rates as reported by IIEF-5 scores did not differ between RU and PU patients.

Minor complications (Clavien-Dindo categories 1-3) were observed in 7 (12%) patients in the redo group (2 wound infection, 2 UTI, 1 hematuria, 1 intra-operative bleeding, 1 catheter sutured resolved with suture absorption, 1 leg neuropathy), none required re-intervention. In the primary group 34 (11%) intra/post-operative complications occurred (p=0.82): 4 wound infection, 22 UTI, 1 trismus, 3 sutured catheters, 4 leg neuropathies. Neither major complications (Clavien-Dindo 4-5) nor re-intervention occurred in either group.

Median follow-up was shorter in the redo group (35 vs. 51 months). This may underestimate very late failures; however, time-to-event analysis and fixed time-point comparisons (e.g., 12-month recurrence) were used to mitigate this. The significantly earlier recurrences in redo cases (median 6 vs. 17 months) remain a sound finding and may reflect more aggressive disease biology, greater spongiofibrosis, or compromised tissue quality from prior interventions (e.g., higher lichen sclerosus prevalence: 12% vs. 5%). Clinically, this underscores the need for vigilant early surveillance in redo patients.

Post-operative follow-up included office visits at 1, 3, 6 and 12 months with uroflowmetry, PVR and USS-PROM. Cystourethroscopy was performed one year post operatively. Routine office cystourethroscopy has been demonstrated to be a poor predictor of functional outcomes (22) in that not all patients with anatomic recurrence on cystoscopy reported symptoms suggesting stricture, therefore further anatomical assessment of the urethra, beyond the cystourethroscopy at 12 months, was limited to patients who reported decline in their urinary flow, either subjectively or objectively by uroflowmetry and PVR parameters or by USS-PROM results (1). It is reasonable to assume that our study was more sensitive for urethral stricture recurrence compared to other studies using less stringent criteria such as only the need for additional treatment. This is in concordance with Erickson et al. who reported lack of clinical recurrence in 35% of those with anatomical stricture recurrence prompting re-treatment (23). We have previously shown that the Peeling voiding diagram that has been incorporated into the USS-PROM is not sensitive enough to pick up reliably on changes in patient uroflowmetry parameters (24). It is thus obvious that simply using avoidance of additional procedures, patient satisfaction and subjective PROM scores, each on its own, is not sufficient to determine urethral stricture recurrence and that a multimodal assessment provides a more accurate outcome evaluation for research purposes. Pre-operative USS-PROM data were incomplete, limiting paired comparisons. Post-operative scores were therefore compared cross sectionally. Bother scores (Q7) were similar between groups. We acknowledge this limitation in the retrospective design. In some instances, a salvage endoscopic procedure after stricture recurrence can offer patients a reasonable outcome. Blaschko et al. reported total success rates rising from 67% to 78% after such procedures (7).

Our study limitations include its retrospective nature, differential follow-up duration, baseline imbalances in stricture location, incomplete pre-operative PROM data, heterogeneity of surgical techniques, and limited statistical power for sub-group analysis. Multivariable adjustment or propensity matching was not performed to avoid overfitting in this modest redo cohort. Future prospective studies with larger samples are warranted.

## CONCLUSIONS

Redo urethroplasty while challenging is feasible with similar recurrence rates to primary cases, though not statistically powered to exclude modest differences; however, stricture recurrence tends to occur earlier after redo surgery. Patient satisfaction after surgery is slightly diminished after repeat urethroplasty, even though patient reported outcomes and bother were similar. These findings support redo urethroplasty as an effective option for recurrent urethral strictures.

## Data Availability

Trial data will be made available on reasonable request to the corresponding author.

## ABREVIATIONS

RU: redo urethroplasty
USS-PROM: Urethral stricture surgery – patient reported outcome measure
PVR: post-void residual
VCUG: voiding cystourethrography
Qmax: maximal flow rate
